# The BMI Z-Score and Protein Energy Ratio in Early- and Late-Diagnosed PKU Patients from a Single Reference Center in Mexico

**DOI:** 10.3390/nu15040957

**Published:** 2023-02-14

**Authors:** Lizbeth Alejandra López-Mejía, Cynthia Fernández-Lainez, Marcela Vela-Amieva, Isabel Ibarra-González, Sara Guillén-López

**Affiliations:** 1 Laboratorio de Errores Innatos del Metabolismo y Tamiz, Instituto Nacional de Pediatría, Secretaría de Salud, Mexico City 04530, Mexico; 2 Unidad de Genética de la Nutrición, Instituto de Investigaciones Biomédicas, UNAM, Mexico City 04510, Mexico

**Keywords:** phenylketonuria, caloric intake, BMI Z-Score, protein intake, newborn screening, rare diseases

## Abstract

The relationship between protein and energy and their appropriate proportions in hyperphenylalaninemia (HPA) or phenylketonuria (PKU) patients in terms of growth have been poorly studied, especially in those diagnosed late. We aimed to describe the protein energy ratio (P:E) and its association with body mass index (BMI) in 638 dietetic and anthropometric assessments from 54 early- or late-diagnosed HPA/PKU patients. Dietetic and anthropometric data were analyzed and classified according to BMI Z-Score and type of diagnosis, early by newborn screening (NBS) or late. Correlation between BMI Z-Score and P:E ratio was established. Percent of dietary protein from Phe-free metabolic formula was analyzed. According to the BMI Z-Score, the majority of assessments were eutrophic (69.4%). The median P:E ratio was >4 in most of the overweight assessments. Remarkably, the underweight group consumed the highest proportion of Phe-free metabolic formula (74.5%). A positive correlation between BMI Z-Score and P:E ratio was found. The highest proportion of underweight was found in the late-diagnosed patients. Our findings might be related to their nutritional history previous to the HPA/PKU treatment. Thus, complex nutritional outcome of the late-diagnosed HPA/PKU patients deserves actions to guarantee the early diagnosis, closer nutritional follow-up and alternative therapeutic approaches.

## 1. Introduction

Phenylalanine hydroxylase (PAH; E.C.1.14.16.1) deficiency is an autosomal recessive inherited defect that results in abnormally high blood levels of the essential amino acid phenylalanine (Phe) and causes a spectrum of hyperphenylalaninemia (HPA), whose most severe form is phenylketonuria (PKU, phenotype MIM number #261600). When PKU is not treated early, it could cause severe encephalopathy that can lead to intellectual disability, epilepsy, behavioral, psychiatric and movement problems, as well as skin and hair hypopigmentation, eczema and musty odor [1]. In this disorder, the treatment requires that Phe must be ingested in limited amounts to maintain a blood level equal to or less than 360 μmol/L during the lifetime of the patient [2] and the benefits of the PKU nutritional treatment has been widely recognized [3]; however, Phe over restriction can lead to malnutrition and poor growth, among other effects. Due to the low intake of Phe and, hence, intact protein, medical or metabolic formulas have been developed to provide energy, vitamins and minerals and higher amounts of protein in a form free of or very limited in Phe [4].

Since its implementation in the 1960s by Robert Guthrie and Ada Susi [5], the newborn screening (NBS) program for HPA/PKU has been established in most high-income countries [6], and has been considered as one of the greatest advances in public health, since early detection of the disease allows prompt intervention to prevent intellectual disability as well as other complications [7]. Unfortunately, NBS is not performed in all countries, especially in those with low and middle income i.e., in Mexico mandatory PKU NBS began in 2011 and its coverage is still partial (close to 84%) [8,9]. Thus, late diagnosis of HPA/PKU patients is still a reality, and nutrition specialists, as well as health care providers, continue facing them [10,11,12]. The age of treatment onset in late-diagnosed HPA/PKU patients is variable [10], thus the nutritional history is very diverse and most of the patients have been exposed to foods with high content of intact protein such as those from animal sources, legumes and nuts [3].

The optimal amounts of total protein, and the proportions of Phe-free metabolic formula and intact protein, in the diet of HPA/PKU patients have been suggested indistinctly for early- or late-diagnosed patients, based on clinical experience and observational studies. However, the protein requirements have been an area of debate [13]. An efficient dietary provision of protein, or any other nutrient, to meet basal demands will only occur when demands for energy are met [14]. Therefore, a safe relationship between protein and energy ratio (P:E) has been established by the WHO for healthy subjects (1.5–2.26 g protein/100 kcal), and safe P:E ratios have been described at different stages in life with specific diets; for example, 1.56–2.14 g protein/100 kcal for infants during the first 55 days of life, fed milk-based infant formulas [15], and 1.8 g protein/100 kcal during the first four months of life, fed infant formula [16,17]. There are limited data supporting a suggested safe P:E ratio for patients with inborn errors of metabolism (IEM), but some authors have suggested a P:E ratio from 1.5 to 2.9 g protein/100 kcal for organic acidemias and urea cycle disorders [18].

In early- or late-diagnosed HPA/PKU patients, an amino acid mix from hydrolysate protein, rather than intact protein, is the primary protein source in the diet, and about 52–80% of total protein comes from this Phe-free metabolic formula [13,19,20]. The utilization of dietary nitrogen from isolated amino acids, like those present in Phe-free metabolic formula, is too rapid compared to intact protein during the postprandial period and requires an adequate intake of energy to support and sustain an anabolic effect [21,22,23]. Thus, it has been suggested that protein requirements should be higher when most of the protein is provided by an amino-acid-based formula to achieve normal growth, due to the rate of protein digestion and the absorption of amino acids, biological value and net protein utilization [24,25,26].

Regardless of the age at treatment onset, HPA/PKU diets need to be well balanced in protein and energy to maintain an adequate metabolic control and avoid under- or overnutrition, and to preserve a normal nutritional status [27]. Some published protocols of estimated protein and energy requirements for HPA/PKU patients vary from 1.21 to 4.58 g protein/100 kcal, depending on the age of the patient [28,29,30,31].

Currently, there is no consensus about the optimal P:E ratio requirements in HPA/PKU patients since there are several guidelines that vary between countries as well as the use and dosage of Phe-free metabolic formula. Additionally, the relationship between protein and energy and their appropriate proportions in terms of growth have been poorly studied. Moreover, there are scarce studies where the P:E ratios of early- and late-diagnosed HPA/PKU patients have been explored and also related to the differences among anthropometric indices. Shakiba et al. found a significantly higher mean Z-Score of head circumference, BMI, height and weight in the NBS-diagnosed group compared with the late-diagnosed group [32]. Thus, the aim of this work was to describe the P:E ratio as well as its influence on body mass index (BMI) in early- and late-diagnosed HPA/PKU patients treated in a reference center in Mexico.

## 2. Materials and Methods

### 2.1. Study Design

**Figure 1:**
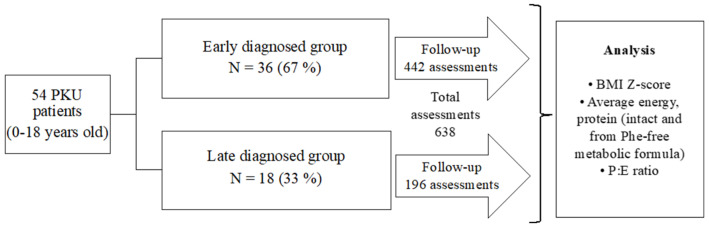
Design of the study.

A general scheme of the study is shown in Figure 1. A longitudinal, observational study was performed in a cohort of Mexican HPA/PKU patients who are regularly treated in an outpatient setting at the Inborn Errors of Metabolism and Screening Laboratory of the National Institute of Pediatrics of Mexico City, which is a tertiary care center specialized in the care of patients with inborn errors of metabolism.

### 2.2. Study Population

Subjects enrolled had the following inclusion criteria: patients from one month to 18 years old who had a diagnosis of PAH deficiency confirmed biochemically (high Phe blood levels and a Phe/Tyrosine (Tyr) ratio >3) and by molecular studies (two demonstrated pathogenic variants in *PAH* [33]; HPA/PKU patients whose nutritional follow-up had been carried out in our reference center, and who had had nutritional treatment with Phe restriction supplemented with Phe-free metabolic formula according to the guidelines of the Southeast Regional Genetics Network (SERN) and the Genetic Metabolic Dietitians International (GMDI) [29]. Patients were categorized into two groups as follows: (1) early-diagnosed patients detected by NBS, whose treatment began in the first month of life and (2) late-diagnosed patients, those who started treatment after the first month of life. Patients with tetrahydrobiopterin (BH_4_) disorders and those under pharmacological BH_4_ therapy (sapropterin) were excluded. The studied subjects did not receive any commercial special low-protein food.

### 2.3. Nutritional Assessment

Caloric and protein intake was assessed using a 3-day 24 h dietary recall. These dietary recalls were analyzed by two experienced metabolic dietitians using a specialized nutrient analysis software (Metabolic Pro program^®^, USA, Genetic Metabolic Dietitians International) [34]. P:E ratio was calculated for each dietary recall of every HPA/PKU patient and expressed as grams of protein/100 kcal. Age at the moment of each assessment was recorded and classified as follows: infants (from 1 month to 2 years), preschoolers (from 2 to 5 years), schoolchildren (from 6 to 10 years) and teenagers (from 11 to 18 years). Anthropometric measurements (weight and height) were performed on a digital pediatric Seca^®^ scale (Hamburg, Germany). For patients younger than two years old, a digital baby Seca^®^ scale (Hamburg, Germany) and a Seca^®^ infantometer (Hamburg, Germany) were used. For patients older than 2 years, a wall-mounted stadiometer and a Seca^®^ scale (Hamburg, Germany) were used. In both groups, weight was recorded to the nearest 0.1 kg and height to the nearest 0.1 cm. Patients were measured with no shoes and wearing underwear. All the measurements were performed by two standardized metabolic dietitians. The product of dividing the weight and squared height was the BMI value. The BMI Z-Score was calculated with Anthro^®^ (Version 3.2.2.1, Geneva, Switzerland) for patients from 0–5 years old, and AnthroPlus^®^ software (Version 1.0.4., Geneva, Switzerland) for individuals from 5–19 years old. Based on this BMI Z-Score, assessments were stratified into 3 groups: underweight (<−1), eutrophic (from −1 to 1) and overweight (>1). Dietary recalls were paired with the BMI Z-Score. Child stunting was defined by a length/height for age Z-Score below −2 standard deviations (SDs) based on the World Health Organization (WHO) [35]. We also classified assessments according to the observed biochemical phenotype of patients, following the three categories established by the highest untreated Phe blood concentration as follows: classical PKU (cPKU; blood Phe > 1200 μmol/L), mild PKU (mPKU; blood Phe 600–1200 μmol/L) and mild hyperphenylalaninemia (MHP; blood Phe 360–600 μmol/L) [36].

### 2.4. Statistical Analysis

Data were analyzed with GraphPad Prism software (Version 9.4.1, San Diego, CA, USA). Normal distribution of data was determined with the Shapiro–Wilk test. Normally distributed data were analyzed with a one-way ANOVA followed by Dunn’s multiple comparisons adjustment. Non-parametric distributed data were analyzed with the Mann–Whitney U test or Friedman test, followed by Dunn’s multiple comparisons adjustment test. The results are expressed as mean ± SD or as median and interquartile range (IQR), for data with parametric and non-parametric distributions, respectively. A *p*-value of <0.05 was considered statistically significant (* *p* < 0.05; ** *p* < 0.01; *** *p* < 0.001; **** *p* < 0.0001). Whether normal or non-parametric distribution, Pearson or Spearman coefficients were calculated to establish the correlation between the BMI Z-Score and P:E ratio.

### 2.5. Ethical Considerations

This study was approved by the institutional research and the biosafety and ethics committees (2021/056 and 2020/014).

## 3. Results

### 3.1. Patient Description

Fifty-four patients were included in the study, 44.4% (24/54) were male and 55.5% (30/54) were female. Thirty-six patients were early diagnosed by NBS (67%), and eighteen (33%) were diagnosed late (Figure 1). The early-diagnosed patients began their treatment before 30 days old, in contrast, patients with late diagnosis began their treatment at an average age of 5 years 6 months, being the youngest 6 months old and the oldest 15 years old.

### 3.2. Nutritional Assessment

A total of 638 three-day dietary recalls were analyzed, with a minimum of two dietary recalls per patient and a maximum of 36. There were 442/638 (69.3%) recalls from NBS-diagnosed patients and 196/638 (30.7%) were from late-diagnosed patients (Figure 1). According to the BMI Z-Score obtained from each assessment, in the NBS group we found that 58/442 (13.1%) were from underweight patients. Eutrophy was found in 322/442 assessments (72.9%), and overweight was found in 62/442 assessments (14%). All the assessments from teenage patients detected by NBS were classified as eutrophic (Table 1). In the late-diagnosed patients, 48/196 assessments (24.5%) were from underweight patients, 121/196 assessments (61.7%) were found eutrophic, and the overweight group included 27/196 (13.9%) assessments.

The height/age Z-Score was also analyzed, and 33/638 (5.17%) were stunted with a Z-Score < −2. Of these, 6/33 (18.1%) were from infants, 22/33 (66.6%) were from preschoolers, 4/33 (12.1%) were from schoolchildren and 1/33 (3%) were from teenagers. None of the height/age Z-Score assessments in the late-diagnosed patients were stunted.

### 3.3. Protein: Energy Ratio

**Figure 2:**
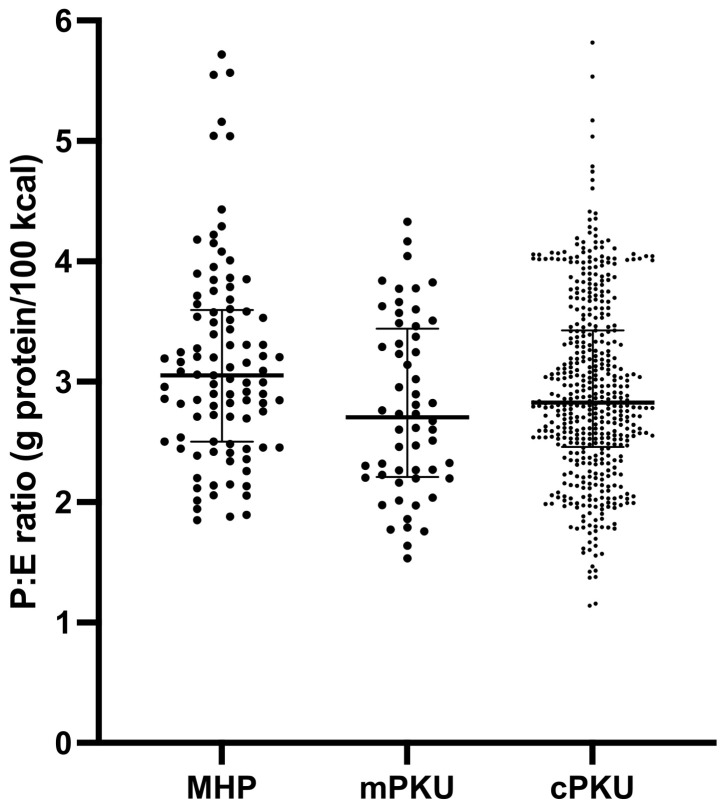
Protein: energy (P:E) ratio of PKU patients according to type of HPA. Patients were classified according to their highest untreated Phe blood concentration as classical PKU (cPKU; blood Phe > 1200 μmol/L), mild PKU (mPKU; blood Phe 600–1200 μmol/L) and mild hyperphenylalaninemia (MHP; blood Phe 360–600 μmol/L) [36]. P:E ratio was calculated from patient’s dietary recalls and is expressed as grams of protein per 100 kcal. P:E ratio is presented as median with 5–95th percentile.

Assessments were classified by HPA/PKU type and P:E ratio was analyzed. There were no statistical differences in the P:E ratio according to the type of HPA/PKU (Figure 2).

**Figure 3:**
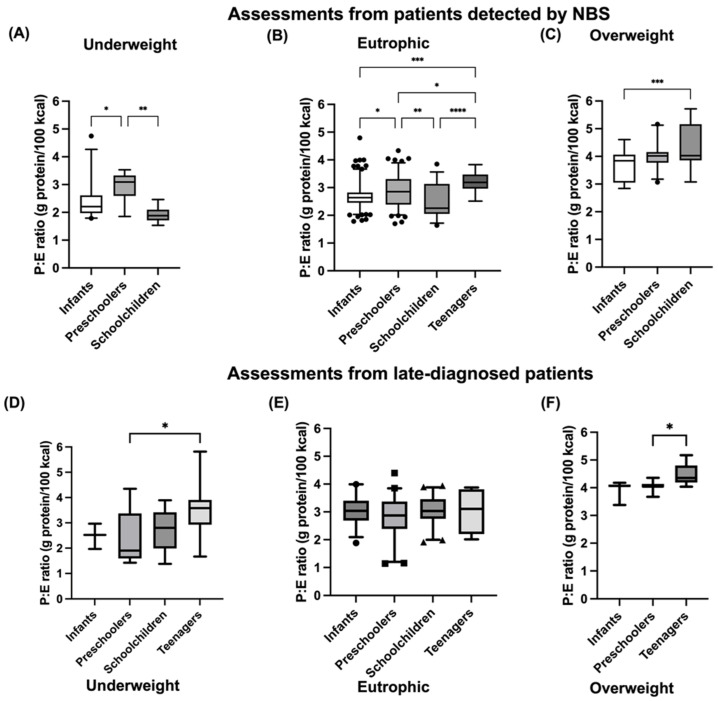
Protein: energy ratio of PKU patients according to age group and BMI Z-Score and type of diagnosis. Classified as underweight, eutrophic and overweight. P:E ratio was calculated from patient’s dietary recalls and is expressed as grams of protein per 100 kcal. P:E ratio is presented as median with 5–95th percentile. (**A**) P:E ratio according to age group and BMI Z- in underweight NBS assessments. (**B**) P:E ratio according to age group and BMI Z- in eutrophic NBS assessments. (**C**) P:E ratio according to age group and BMI Z- in overweight NBS assessments. (**D**) P:E ratio according to age group and BMI Z- in underweight late-diagnosed assessments. (**E**) P:E ratio according to age group and BMI Z- in eutrophic late-diagnosed assessments (**F**) P:E ratio according to age group and BMI Z- in overweight late-diagnosed assessments. Statistical differences between the studied age groups are shown. A *p*-value *<* 0.05 was considered statistically significant (* *p <* 0.05, ** *p <* 0.01, *** *p <* 0.001, **** *p <* 0.0001). Nutritional status was assessed according to BMI Z-Score. Age groups: infants (<2 years), preschoolers (2 years to <6 years), schoolchildren (6 to <11 years) and teenagers (11 to 18 years).

Figure 3 depicts the P:E ratio according to age group and BMI Z-Score classification for both NBS- and late-diagnosed patients. Remarkably, compared with underweight and eutrophic assessments, in both groups of overweight (NBS- and late-diagnosed) regardless of the age, the median of the P:E ratio was >4 (Figure 3C,F). In Table S1, the medians with 25th and 75th percentiles are presented in all groups by type of diagnosis (NBS- or late-diagnosed), and according to age and BMI Z-Score classification (Table S1).

### 3.4. Protein Intake Proportion between Intact Protein and Metabolic Formula

**Figure 4:**
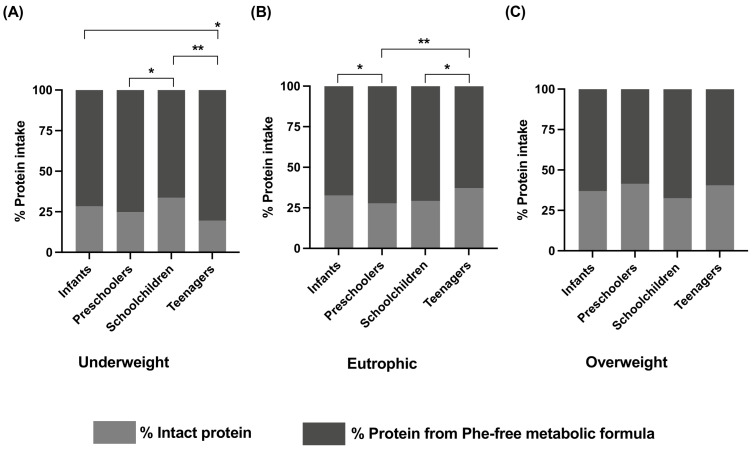
Percentage of protein intake of PKU patients classified by age and nutritional status. The percentage of protein intake from natural protein and from PHE-free nutritional formula was calculated from patients’ dietary recalls. (**A**) Percentage of protein intake from Phe-free metabolic formula and intact protein in underweight patients. (**B**) Percentage of protein intake from Phe-free metabolic formula and intact protein in eutrophic patients. (**C**) Percentage of protein intake from Phe-free metabolic formula and intact protein in overweight patients. Statistical differences in the percentage of protein intake from PHE-free nutritional formula between the studied age groups are shown. A *p*-value < 0.05 was considered statistically significant (* *p* < 0.05, ** *p* < 0.01. Nutritional status was assessed according to the BMI Z-Score. Age groups: infants (<2 years), preschoolers (2 years to <6 years), schoolchildren (6 to <11 years) and teenagers (11 to 18 years).

The percentage of protein intake from Phe-free metabolic formula and intact protein is shown in Figure 4. In underweight patients (Figure 4A), the highest percentage of the Phe-free metabolic formula was consumed by the teenagers, with a median of 83% (77–86.8), followed by preschoolers, with a median of 76.2% (70.9–78.7), infants, with a median of 72.9% (66.4–76.9), and schoolchildren, with a median of 70.2% (56.2–80.5). Significant differences were observed between schoolchildren and both preschoolers (*p* < 0.05) and teenagers (*p* < 0.01). Conversely, in the eutrophic group (Figure 4B), the teenagers consumed the lowest percentage of protein from Phe-free metabolic formula, with a median of 62.5% (53.1–77), and schoolchildren had the highest percentage with a median of 75.7% (61.3–80.8). A median of 73.7% (64.4–79.7) was observed in preschoolers, and 70.3% (60–75.5) was found in infants. The most significant difference was observed between preschoolers and teenagers (*p* < 0.01). In the overweight group (Figure 4C), schoolchildren also consumed a greater amount of protein from Phe-free metabolic formula, with a median of 70.3% (58.9–75.3). Preschoolers ingested the lowest percentage of protein from Phe-free metabolic formula with 62% (49.7–70.2), while infants and adolescents consumed similar amounts, with a median of 65.1% (58.6–69.5) and 65.5% (41.2–74.5), respectively. In this overweight group, no significant differences were found.

**Figure 5:**
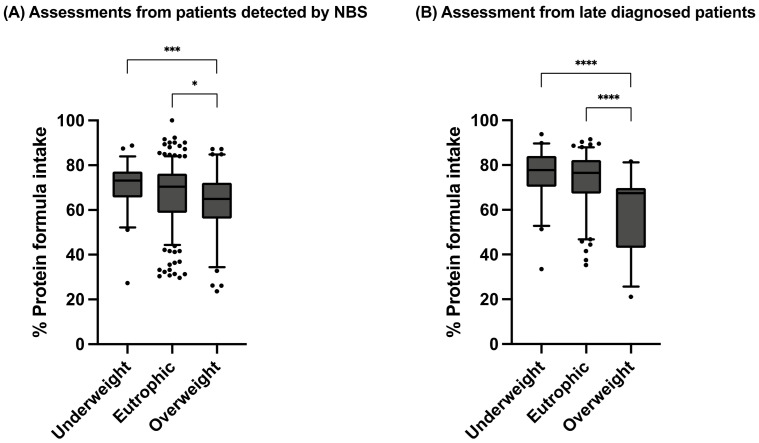
Percentage of protein intake from Phe-free nutritional formula in assessments from dietary recalls of PKU patients. (**A**) Assessments from patients detected by NBS. (**B**) Assessments from late-diagnosed patients. P:E ratio is presented as median with 5–95th percentile. Statistical differences between the studied age groups were calculated. A *p*-value < 0.05 was considered statistically significant (* *p* < 0.05, *** *p* < 0.001, **** *p* < 0.0001). Nutritional status was assessed according to BMI Z-Score. Age groups: infants (<2 years), preschoolers (2 years to <6 years), schoolchildren (6 to <11 years) and teenagers (11 to 18 years).

The percentage of protein from Phe-free metabolic formula was also analyzed according to the BMI Z-Score classification, considering all assessments, regardless of their age, by type of diagnosis (Figure 5). Regardless of the diagnosis type, the overweight group was the one that consumed the lowest percent of protein from Phe-free metabolic formula. Moreover, we found that the overweight group was statistically different to underweight and eutrophic groups.

**Figure 6:**
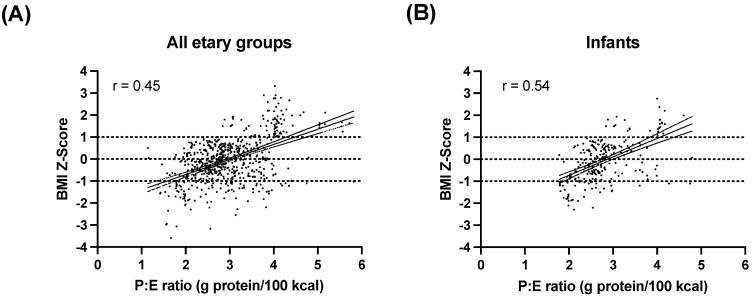

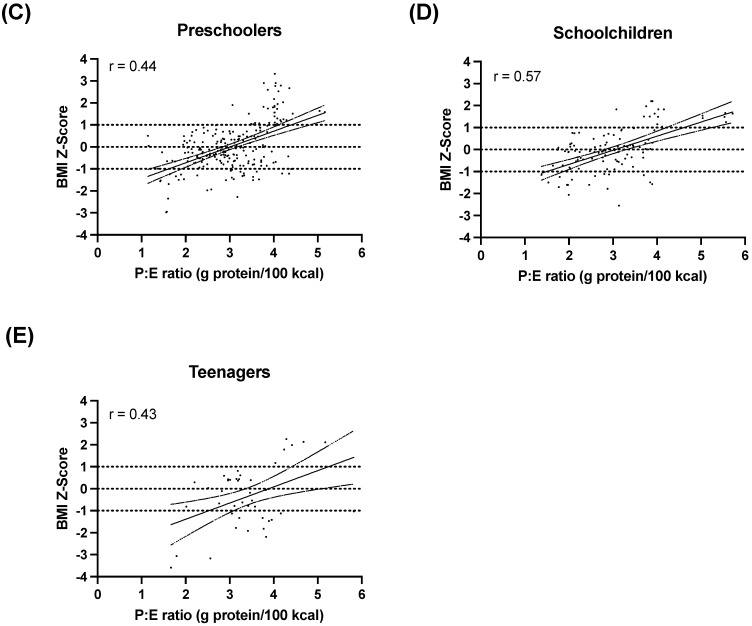
Pearson’s correlation between P:E ratio and BMI Z-Score in PKU patients classified by age group. P:E ratio was calculated from patients’ dietary recalls and is expressed as protein per 100 kcal. BMI Z-Score was calculated from anthropometric measurements (weight, height). Eutrophic state (BMI Z-Score between −1 and 1) is indicated in dotted lines. (**A**) Correlation between the BMI Z-Score and the P:E ratio in all etary groups. (**B**) Correlation between the BMI Z-Score and the P:E ratio in infants (<2years). (**C**) Correlation between the BMI Z-Score and the P:E ratio in preschoolers (2 years to <6 years). (**D**) Correlation between the BMI Z-Score and the P:E ratio in schoolchildren (6 to <11 years). (**E**) Correlation between the BMI Z-Score and the P:E ratio in teenagers (11 to 18 years).

The correlation between the BMI Z-Score and the P:E ratio was investigated in all anthropometric assessments by age group. In all cases, a positive correlation between these two factors was observed (Figure 6).

## 4. Discussion

The present study describes the relation between P:E ratio and the BMI Z-Score as well as the total protein intake, from both intact protein and Phe-free metabolic formula, in 638 nutritional assessments from the longitudinal follow-up of 54 HPA/PKU patients, who were diagnosed early or late. To the best of our knowledge, this is the first and largest published cohort of PKU patients from Mexico in which the P:E ratio and BMI Z-Score were studied. One strength of our study is the homogeneous PAH-deficient cohort and the large number of dietary recalls included (638) and also all of the patients had a sufficient and constant amount of Phe-free metabolic formula. The major finding in our study was the positive correlation between the BMI Z-Score and P:E ratio in all age groups, regardless of the type of diagnosis (Figure 6). Another important finding is the anthropometric differences among NBS-diagnosed patients compared to those diagnosed late.

Similar to other studies [37,38], we found a high proportion of eutrophic anthropometric assessments in the NBS group (72.9%). Moreover, this proportion was slightly smaller in the late-diagnosed group (61.7%).

In the general Mexican population, obesity is a public health problem, especially in the pediatric population, where 37.4% of schoolchildren and 42.9% of teenagers are overweight [39,40]. In the present study, we found approximately half of these overweight proportions for these age groups. However, analyzed by type of diagnosis, schoolchildren of the NBS group presented the highest percentage of overweight assessments with 31% (Table S2). These data of overweight in schoolchildren are similar to those previously reported by Scaglioni et al. that showed an association between BMI rebound and overweight at 8 years in HPA patients [41]. In Mexico, there are different factors associated with overweight in childhood, such as: high consumption of sugary drinks, sedentary activities and a diet rich in fat and carbohydrates [42]; a recent systematic review of overweight in PKU children also found a high caloric intake and lack of physical activity as possible causal factors [43]. Moreover, it is known that obesity represents one of the most frequent comorbidities in HPA/PKU patients [44,45,46], which indicates the necessity of a closer nutritional follow-up and new therapeutic approaches. Overweight in this study occurred in spite of the lack of commercial special low-protein foods, although there is still not sufficient evidence of its direct impact. Excessive consumption would lead to unnecessary energy intake since these products have 75% more energy content than regular foods [47].

Herein, a high proportion of underweight patients in all age groups was observed compared to the BMI Z-Score of the general Mexican population [40,48] (Table S2). While in the general population 1.5% of infants and preschoolers are underweight, we found a 10-fold higher percentage (15.2%). Additionally, for the schoolchildren group we found 16.7% and the highest percentage found in our study was from the teenagers with 31.8%. It is important to highlight the small number of assessments that we had of teenagers compared to the other age groups. The presence of undernutrition in our study might be attributable to multiple factors, i.e., in Mexico undernutrition is a consequence of poverty and disparities in health services for socially disadvantaged populations [49]. Regarding PKU and the special diet, underweight could be attributable to the rapid metabolism of the free amino acids contained in the Phe-free metabolic formula [21,26], which indicates the need for a higher energy prescription (Figure 4A).

Interestingly, the highest proportions of underweight assessments were observed in the late-diagnosed patients. This might be related to the nutritional history of these patients, who, previous to the diagnosis, ingested high-intact-protein foods, which became a difficult issue when establishing the Phe-restricted diet. We would have expected difficulty accepting the proper quantities of Phe-free metabolic formula frequently observed in late-diagnosed patients who are neurologically impaired as reported [50], but instead all late-diagnosed HPA/PKU patients, underweight, eutrophic and overweight, had nearly the same percentages of protein intake from Phe-free metabolic formula (Figure 5). In this group of patients, the use of commercial special low-protein foods could be a good complementary therapeutic alternative to improve the energy intake as previously described as these products are mainly used to provide energy and variety to the Phe-restricted diet [27]. Unfortunately, in Mexico as in other low- or middle-income countries, the availability of these type of foods is scarce [51].

A higher proportion of underweight was observed in the late-diagnosed patients (18.5%) compared with the NBS-diagnosed patients (14.1%) (Table S3). Shakiba et al. also found a similar tendency of underweight in their late-diagnosed patients, compared with their NBS HPA/PKU group [32].

Taken together, these findings show the necessity to strengthen educational tools for families coping with HPA/PKU and help them prevent suboptimal nutrition. Additionally, the necessity for optimization and universalization of the NBS programs is evidenced [12].

Stunting prevalence in all age groups was 5.17% (33/638). Considering the 223 assessments from preschoolers, 9.8% (22/223) were the most affected in terms of height/age Z-Score. This percentage is slightly lower than the estimated percentage of preschoolers with stunting according to a national survey for healthy children (12.6%) [40]. Our finding is consistent with other studies on PKU patients that have reported subjects to be significantly shorter for their age compared to a reference population in the first years of life, mainly attributable to the use of a Phe-restricted diet with the use of a Phe-free metabolic formula [52,53]. Contrary to other reported data on PKU [32], all of our studied stunted group was diagnosed through NBS. Interestingly, Thiele et al. found a significantly reduced growth rate during the first 2 years of life in PKU patients and in puberty [54], this differs from our study in which the most affected group was from 2 to 5 years.

Our study shows a P:E ratio of 2–4 g protein/100 kcal in relation to optimal growth (Figure 3 and Figure 6) regardless of the type of diagnosis, including intact protein as well as protein from Phe-free metabolic formula, in all of our patients. This is approximately 8–16% of the total energy intake as protein. The percentages of these two sources of protein intake varied, as shown in Figure 4. Meanwhile, Evans et al. reported a slightly higher safe P:E ratio, between 3 and 4.5 g protein/100 kcal/d, which correlated with optimal growth [30]. In our results, we found a P:E ratio above 4 to be related to overweight, while other studies report as safe a P:E ratio higher than 4. Thus, further studies are needed to have a better-defined safe interval to prevent suboptimal growth in HPA/PKU patients.

Our data showed that 70–83% of the protein came from Phe-free metabolic formula, as shown in Figure 4 and Figure 5. It is known that the amino acids in Phe-free metabolic formula have a different dietary nitrogen postprandial utilization rate than intact protein [21,22,23,24], and this could have further repercussions on energy demand and on the P:E ratio.

The importance of the prescription of a correct P:E ratio has also been highlighted by several recent studies investigating optimal oral/enteral feeds for catch-up growth. In these studies, it is suggested that, in children experiencing faltering growth, it is necessary to increase energy, protein and micronutrients [55]. An accurate estimation of the protein requirement from metabolic formula in HPA/PKU patients is especially important in low- and middle-income countries, in which Phe-free metabolic formula is scarce and costly [51,56]. Recommending an unnecessarily high P:E ratio may contradict PKU recommendation guidelines and require excessive and expensive protein substitutes, including metabolic formula [57]. Moreover, renal dysfunction has been observed in HPA/PKU patients treated with relatively high protein intake from synthetic amino acid formula [58,59].

The use of 24 h dietary recalls over three days allows for an actual representation of the daily diet protein and energy intake instead of the dietary prescription. Additionally, the use of a specialized software (Metabolic Pro^®^) [34] allowed us to perform a uniform analysis of the data and to systematize protein and energy quantification.

An important limitation in this study is the lack of body composition studies in our patients, since BMI has limitations as a measure of total body lean mass and fat mass, especially in underweight children [60]. Another limitation is the well-known bias implied by self-reported dietary recalls. A three-day recall may not be representative of the usual diet, and patients can intentionally misreport their consumption of certain foods [61]. Finally, it is important to mention the biased selection of the study population, since the inclusion criteria included patients who were able to complete the three-day dietary recall, which resulted in a close nutritional follow-up and an improved nutritional status.

## 5. Conclusions

In the present study, a P:E ratio between 2 and 4 was associated with a normal BMI Z-Score regardless of the type of diagnosis. The majority of the overweight group had a P:E ratio above 4, while no clear relationship was found in the undernourished group, so further studies in this latter group are needed. In this study, the highest percentage of overweight PKU NBS-diagnosed patients was found in the schoolchildren, and the highest percentage of underweight was found in the late-diagnosed teenagers, and further studies are needed in order to improve nutritional status in this specific group. Taken together, our results suggest not only that protein intake should be calculated, but so should its relationship with energy, because it appears to be related to nutritional status. Diets for HPA/PKU patients, especially for those diagnosed late, need to be well balanced in protein and energy if an adequate BMI Z-Score is to be maintained.

## Figures and Tables

**Table 1:** Assessments from HPA/PKU patients by type of diagnosis, age and BMI Z-Score classification.

Assessment from NBS Diagnosed Patients (*n* = 442)					
	Infants *n* (%)	Preschoolers *n* (%)	Schoolchildren *n* (%)	Teenagers *n* (%)	**TOTAL (%)**
**Underweight**	39 (67.2)	13 (22.4)	6 (10.3)	0 (0)	**58 (13.1)**
**Eutrophic**	160 (49.7)	111 (34.5)	34 (10.6)	17 (5.3)	**322 (72.9)**
**Overweight**	19 (30.6)	25 (40.3)	18 (29)	0 (0)	**62 (14.0)**
**Assessment from late-diagnosed patients (*n* = 196)**					
	Infants *n* (%)	Preschoolers *n* (%)	Schoolchildren *n* (%)	Teenagers *n* (%)	**TOTAL (%)**
**Underweight**	3 (24.5)	18 (37.5)	13 (27.1)	14 (29.2)	**48 (24.5)**
**Eutrophic**	28 (61.7)	43 (35.5)	43 (35.5)	7 (5.8)	**121 (61.7)**
**Overweight**	8 (13.8)	13 (48.1)	0 (0)	6 (22.2)	**27 (13.9)**

## Data Availability

The original contributions presented in the study are included in the article, further inquiries can be directed to the corresponding author.

## Supplementary Materials

The following supporting information can be downloaded at: https://www.mdpi.com/article/10.3390/nu15040957/s1, Table S1: Median and percentile values of P:E ratio from HPA/PKU patients detected by NBS or late-diagnosed. Table S2 [62]: BMI Z-Score of general Mexican population and of HPA/PKU patients. Table S3: BMI Z-Score of HPA/PKU patients by type of diagnosis.

## Abbreviations

BMI	body mass index
HPA	hyperphenylalaninemia
PKU	phenylketonuria
P:E ratio	protein and energy ratio
PAH	Phenylalanine hydroxylase
Phe	phenylalanine
WHO	World Health Organization
IEM	inborn error of metabolism
Tyr	tyrosine
SERN	Southeast Regional Genetics Network
GMDI	Genetic Metabolic Dietitians International
BH_4_	tetrahydrobiopterin
cPKU	classical PKU
mPKU	mild PKU
MHP	mild hyperphenylalaninemia
IQR	interquartile range
